# Optimizing Pectin Yield From Burmese Grape (*Baccaurea ramiflora*) Peels Using Box–Behnken Design and Quality Evaluation

**DOI:** 10.1155/2024/8064657

**Published:** 2024-12-19

**Authors:** Md Akram Hossain, Md. Masud Rana, Mir Tuhin Billah, Md Moinul Hosain Oliver, M. Amdadul Haque

**Affiliations:** ^1^Department of Food Processing and Preservation, Faculty of Engineering, Hajee Mohammad Danesh Science & Technology University (HSTU), Dinajpur 5200, Bangladesh; ^2^Department of Agro-processing, Faculty of Agriculture, Bangabandhu Sheikh Mujibur Rahman Agricultural University (BSMRAU), Gazipur 1706, Bangladesh; ^3^Department of Food Engineering and Technology, Faculty of Engineering, Hajee Mohammad Danesh Science & Technology University (HSTU), Dinajpur 5200, Bangladesh; ^4^Department of Agricultural Engineering, Faculty of Agriculture, Bangabandhu Sheikh Mujibur Rahman Agricultural University (BSMRAU), Gazipur 1706, Bangladesh

**Keywords:** *Baccaurea ramiflora* peel, Box–Behnken Design, optimization, pectin extraction, response surface methodology

## Abstract

Pectin, a polysaccharide, is widely used as a gelling and thickening agent in the food industry. This study undertook optimization of pectin extraction from the peels of *Baccaurea ramiflora* Lour. (Burmese grape or lotkon), an abundantly grown wild fruit in Bangladesh. We applied the Box–Behnken Design of Response Surface Methodology with varied processing parameters including pH, time, and temperature. The Response Surface Methodology, employing a second-order polynomial model, successfully optimized the extraction conditions for the maximum pectin yield. The model predicted a pectin yield of 10.73%, which closely matched the experimental yield of 10.56%. The optimal conditions for pectin extraction were determined as pH (2.4), extraction time (56 min), and temperature (76°C). Further analyses of the extracted pectin under optimized conditions confirmed its excellent potential for food applications. The pectin was characterized by its moisture content (10.42%), water activity (0.51), ash content (3.41%), equivalent weight (769.23 mg/mole), methoxyl content (7.75%), anhydrouronic acid content (66.88%), degree of esterification (65.79%), and acetyl value (0.39%). These determined parameters strongly support that the pectin extracted from the peels of Burmese grapes is of good quality.

## 1. Introduction

Global fruit waste accounts for nearly 42% of the total food waste [1]. The wastes are being generated across multiple stages of production [2], processing [3], and consumption [4, 5]. Fruit wastes are often needlessly discarded as waste even though they are rich in different bioactive compounds [6], essential oils [7], phytochemicals [8], dietary fiber, and pectin [9], among others. With proper protocols in place, these important compounds can be safely harvested from the fruit waste creating further value for the industry. Moreover, the fruit wastes often end up in landfills and contribute to global greenhouse gas (GHG) emissions [10]. Processing fruit waste, particularly pomace, peels, and seeds, can be used for extracting valuable compounds or enriching food items, thereby helping to reduce the carbon footprint of the food and allied industries [11–13].

**Figure 1:**
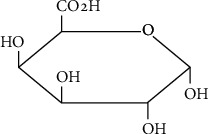
Chemical structure of pectin.

Numerous agricultural by-products and fruit wastes have been investigated [14] for their potential as a source of different bioactive compounds and chemicals. Among the compounds, pectin's global demand is increasing fast, and it has received much attention from the scientific community as well [15]. Pectin sources that have been reported in the literature include banana peel [16, 17]; watermelon rind and pomegranate peels [18, 19]; apple, orange, green citron, and pomegranate peels [20]; mandarin peels [15]; dragon fruit peels [21]; jackfruit waste [22, 23]; guava pomace [24]; soy hull [25]; sunflower residues ([26]; ambarella peels [27]; cocoa bean husks [28, 29]; pomelo peels [30]; citron peels [31]; passion fruit peels [32, 33]; and mango peels [34]. Pectin is a polysaccharide that is present in plant cell walls. It primarily comprises a complex mixture of d-galacturonic acid monomers (Figure 1).

It has a wide range of applications (i.e., gelling, adsorption, thickening, emulsification, and stabilization) in the food, pharmaceutical, and cosmetic industries [16, 35–37]. The quality and use of pectin, however, varies depending on the source and methods of extraction. Traditionally, apple pomace and citrus peels have been the main sources of commercial pectin [38, 39]. In order to keep up with the growing demand, it is important to explore other inexpensive and feasible sources as reported in this manuscript.

*Baccaurea ramiflora* Lour. commonly known as Burmese grape or lotkon locally (in the South Asian region), is an edible fruit belonging to the Euphorbiaceae family. This golden-yellow coloured fruit remains underutilized and underexploited, although it is rich in bioactive compounds [40]. The fruit comprises three main parts: peel, pulp, and the seeds. The tiny pulp portion, known for its medicinal and health benefits, has been consumed since ancient times [41–43]. The remaining inedible peel and the seed, constituting almost 55.5% of the fruit, usually go to the bin. The peel of lotkon is a rich source of bioactive compounds, nutrients, and pectin [44–46]. These peels have been utilized for the extraction of antioxidants [40], preparation of biochar for the treatment of wastewater [47], and other purposes. Although several efforts have been made, the scientific community has rarely invested in the extraction of pectin from lotkon peels. This study is aimed at contributing to this area of research and reporting optimal protocols for pectin extraction, and characterization.

## 2. Materials and Methods

### 2.1. Materials

The study was carried out at the Department of Agro-Processing, Bangabandhu Sheikh Mujibur Rahman Agricultural University (BSMRAU), Bangladesh. A few of the laboratory analyses were performed at the Post-Harvest Technology Division of the Bangladesh Agricultural Research Institute (BARI), Gazipur, Bangladesh. All chemicals and solvents used in the research were of analytical grade. Fresh and mature Burmese grapes (lotkon) were procured from local markets and orchards in Gazipur and Narsingdi districts during the rainy season (August-September). The fruits were transported to the laboratory in cushioned plastic crates to prevent transport injury. The fruits were washed with clean water to remove adhered dirt and placed on a workbench for air drying and further processing.

### 2.2. Preparation of Burmese Grape (Lotkon) Peel Powder

First, healthy and mature Burmese grape (lotkon) fruits, identified by their lack of bruises or impact spots and their bright yellowish skin color, were selected and washed with clean water to remove dirt and soil. Subsequently, the peel and the pulp were separated. The peels were then sliced and dried at 60°C for 36 h in a cabinet drier. Following the drying process, the peels were transformed into powder using a high-shear blender (Jencons Lab.). This powder underwent sieving with a stainless-steel sieve (Sieve no. MIC-300) and was stored in a glass jar at room temperature for subsequent use.

### 2.3. Extraction of Pectin from the Peel Powder

**Figure 2:**
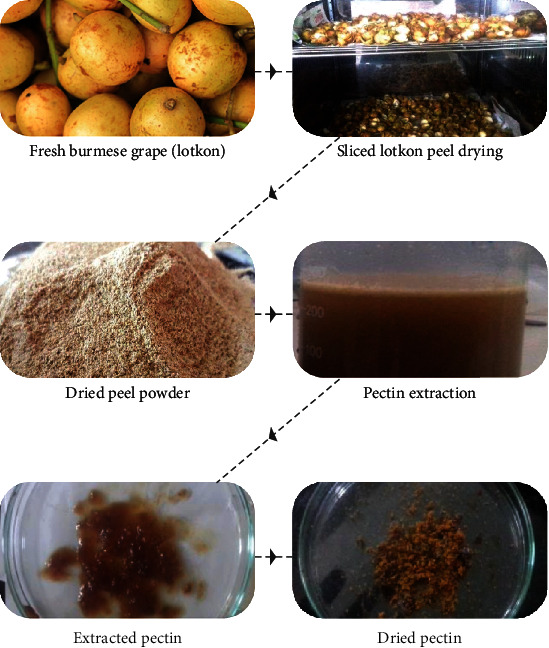
Overall approach of pectin extraction from Burmese grape (lotkon) peel.

The overall approach for pectin extraction was adopted from Liew, Chin, and Yusof [32] with slight modifications, highlighted in Figure 2.

The solid–liquid pectin extraction method in an acidic solvent was used and the conditions (pH, time, and temperature) were followed as defined by the Box–Behnken Design (BBD) as mentioned in Table 1.

A 10 g of lotkon peel powder was mixed with 250 mL of water (1:25 w/v); then, pH was adjusted using 0.1 N HCl. The mixture was heated to the defined temperatures times in a temperature-controlled water bath. A thermometer was inserted into each of the samples to observe the temperature. After heating, the solution was filtered and pressed to recover the extract. Pectin was then precipitated with the addition of ethanol (95%) in a 1:1 ratio (1 part extract and 1 part ethanol) and left overnight at room temperature. Using Whatman No. 1 filter paper, the precipitated pectin was filtered and then dried in a cabinet drier at 60°C for 24 h. The extracted lotkon peel pectin was stored in a glass bottle at room temperature for further analysis. Pectin yield was calculated using Equation (1).

(1) $$\begin{array}{c} \text{Pectin yield}\left(\%\right)=\left(\frac{\text{Weight of dried pedctin }}{\text{Weight of dried peel powder used for extraction }}\right)\times 100 \end{array}$$

### 2.4. Physiochemical Analysis

Moisture and ash analyses of lotkon peel powder and extracted pectin were conducted using methods outlined in AOAC [48]. Moisture content was determined through oven drying and ash content through a muffle furnace. The water activity was also determined using a water activity meter (Aqualab 3 analyzer, Decagon Devices, Pullman, WA).

### 2.5. Characterization of Pectin

The dried pectin extracted from the peel following the optimal conditions was subjected to various quantitative and qualitative tests for characterization.

#### 2.5.1. Determination of Equivalent Weight

The determination of the equivalent weight of the pectin was carried out by following the procedure outlined by Ranganna [49]. Initially, a 0.5-g pectin sample was placed in a 250-mL conical flask. Then, 5 mL of ethanol, 1 g of sodium chloride, 100 mL of distilled water, and 6 drops of phenol red were added to the conical flask. The solution was titrated against 0.1 N sodium hydroxide until a purple color marked the end point. This neutralized solution was then stored for the determination of methoxyl content. Equivalent weight was calculated by following Equation (2).

(2) $$\begin{array}{c} \text{Equivalent weight }\left(\text{mg}/\text{mole}\right)=\left(\frac{\text{Weight of sample}\times 1000}{\text{mL of Alkali}\times \text{Normality of Alkali }}\right) \end{array}$$

#### 2.5.2. Determination of Methoxyl Content (MeO)

The procedure to determine methoxyl content aligned with the method described by Ranganna [49]. Following the determination of the equivalent weight, the neutral solution was retained. Subsequently, 25 ml of 0.25 N sodium hydroxide was added to the solution, thoroughly stirred, and left at room temperature for 30 mins. Then, 25 ml of 0.25 N hydrochloric acid was introduced and titrated against 0.1 N NaOH until the end point, marked by a purple color. Methoxyl content was calculated using Equation (3), where the value 31 in the equation represents the molecular weight of the methoxyl group.

(3) $$\begin{array}{c} \text{Methoxyl content }\left(\%\right)=\left(\frac{\text{ml of Alkali}\times \text{Normality of Alkali}\times 31}{\text{Weight of sample}\times 1000}\right)\times 100 \end{array}$$

#### 2.5.3. Determination of Total Anhydrouronic Acid Content (AUA)

Total AUA of pectin was calculated using the following (Equation 4) [19]:

(4) $$\begin{array}{c} \text{Anhydrouronic acid }\left(\%\right)=\left(\frac{176\times 0.1\;z\times 100}{w\times 1000}\right)+\left(\frac{176\times 0.1\;y\times 100}{w\times 1000}\right) \end{array}$$

Here, the molecular unit of AUA (1 unit) equals 176 g. In the equation, “*z*” represents the volume (in milliliter) of NaOH from the equivalent weight determination, “*y*” represents the volume (in milliliter) of NaOH from the methoxyl content determination, and “*w*” represents the weight of the sample.

#### 2.5.4. Determination of Degree of Esterification (DE)

The DE was determined following the method outlined by Silva et al. [50]. Initially, 20 mg of dried pectin was moistened with ethanol and dissolved in 20 mL of deionized water at 40°C. The solution was combined with phenolphthalein indicator and titrated with 0.5 M NaOH until the color change occurred. The volume of NaOH solution used was noted as the initial titer (*V*1).

Subsequently, 10 mL of 0.5 M NaOH was introduced to the solution, vigorously mixed, and left to stand for 15 min. Following this, 10 mL of 0.5 M HCl was added, and the solution was agitated until the pink color disappeared. The solution was then titrated with 0.5 M NaOH, and the volume used was recorded as the saponification titer or final titer (*V*2). The DE was computed using the following (Equation 5):

(5) $$\begin{array}{c} \text{DE}=\left(\frac{V2}{V1+V2}\right)\times 100 \end{array}$$

where *V*_1_ is the initial titer value and *V*_2_ is the final titer value.

#### 2.5.5. Determination of Acetyl Value

The determination of the acetyl value, following the procedure outlined by Ranganna [49], utilized Equation (6). Initially, 0.5 g of pectin was mixed with 25 mL of 0.1 M NaOH, dissolved by stirring, and left to stand overnight. These mixtures were then diluted to 250 mL using deionized water. A 20 mL aliquot was introduced into the distillation apparatus along with 20 mL of magnesium sulfate-sulfuric acid solution (prepared by mixing 100 g of magnesium sulfate with 1.5 g of sulfuric acid and diluting to 180 mL) and distilled to collect approximately 100 mL of distillate. The distillate was titrated with 0.5 M NaOH (0.05 M) using a phenol red indicator. The value 4.3 in the equation represents the molecular weight equivalent of the acetyl group.

(6) $$\begin{array}{c} \text{Acetyle value }\left(\%\right)=\frac{\text{mL of Alkali}\times \text{Normality of Alkali}\times 4.3}{\text{Weight of sample in aliquot}} \end{array}$$

#### 2.5.6. Determination of Color

The color measurement procedure was conducted using a colorimeter, in line with the method outlined by Ansorena et al. [51]. The surface color of the samples was assessed employing a handheld colorimeter (CM2500d, Konica, Minolta Optics Inc., Japan) based on the CIE Lab^*^ color space. The *L*^∗^ values signify brightness, *a*^∗^ corresponds to the red-green color spectrum, and *b*^∗^ indicates the yellow-blue color spectrum. Each sample underwent three measurements.

### 2.6. Statistical Analysis

Each physiochemical analysis was performed in triplicate for every sample. The results are presented as mean values accompanied by the standard deviation. Statistical analysis of the data was conducted using SPSS v25 (IBM, United States).

### 2.7. Experimental Design Using BBD

The study employed a BBD of Response Surface Methodology (RSM) to evaluate the effect of three key variables: pH (2–3), extraction time (20–60 mins), and temperature (60°C–100°C) on a response variable, pectin yield. Table 1 summarizes the ranges and levels of the three independent variables. This design resulted in 15 experimental runs, including three center points, where all variables were set at their intermediate levels. The experimental data were fitted to a second-order polynomial model, which accurately captured the relationship between the independent variables (pH, extraction time, and temperature) and the response variable (pectin yield). The model's suitability was rigorously assessed through analysis of variance (ANOVA) and diagnostic plots. Numerical optimization techniques, coupled with Derringer's desirability function methodology, were employed to determine the optimal process conditions for maximizing pectin yield. Additional experiments were conducted to verify the predictive accuracy of the optimized response. The experimental design and optimization were performed using the Design-Expert trial Version 12.0.0 (Stat-Ease Inc., Minneapolis, United States).

## 3. Results and Discussion

### 3.1. Pectin Yield

Table 2 presents the pectin yield under various experimental conditions derived by BBD. Pectin yield ranged from 5.40% to 10.45% which is consistent with the findings of Mohanasundaram et al. [52], who reported pectin yield from prickly pear in the range of 2.30% to 10.00%. These values were higher than the pectin content of wild cardamom peel (3.09%) [53] and lower than apple pomace (26.09%) [54].

### 3.2. Model Fitting and Statistical Analysis

The RSM program was employed to determine the most fitting model using the experimental values (pH, time, temperature and pectin yield). The program identified the quadratic model as the best fit, supported by a significant *p* value (*p* < 0.001), high *R*^2^ values (> 0.90), and an insignificant lack of fit (Table 3). Based on this model, a second-order polynomial equation incorporating linear, interactive, and quadratic terms was established. This equation helps explain the relationship between the independent and response variables. The coded factor with the intercept is expressed in Equation (7) below.

(7) $$\begin{array}{c} \text{Pectin Yield }\left(\%\right)=9.36-0.51\text{A}+1.63B-0.39C-0.12\text{AB}-0.11\text{AC}-0.02\text{BC}-1.80A2-0.04\text{B}2-1.06C2 \end{array}$$

where *A*, *B*, and *C* represent pH, time, and temperatures, respectively.

The significance of the model was evaluated through ANOVA, as detailed in Table 3, showcasing a high *F* value (44.09) at *p* < 0.001, indicating the strength of the model in explaining the variation in the response. The “goodness of fit” was assessed through various metrics, including *R*^2^ (0.99), adj-*R*^2^ (0.97), predicted-*R*^2^ (0.89), and CV (coefficient of variance). The high *R*^2^ value reflects the precision of the model in illustrating the relationship between independent and response variables. The balanced adj-*R*^2^ and pre-*R*^2^ values indicate a good correlation. A low coefficient of variance (4.06) signifies the precision and reliability of the conducted experiments. Adequate precision (> 4) further confirms the accuracy of the designed model. The lack of fit *F* value (0.5933) with a high *p* value (0.6769) indicates that the model is suitable for predicting the response.

### 3.3. Effect of the Extraction Conditions on the Pectin Yield

**Figure 3:**
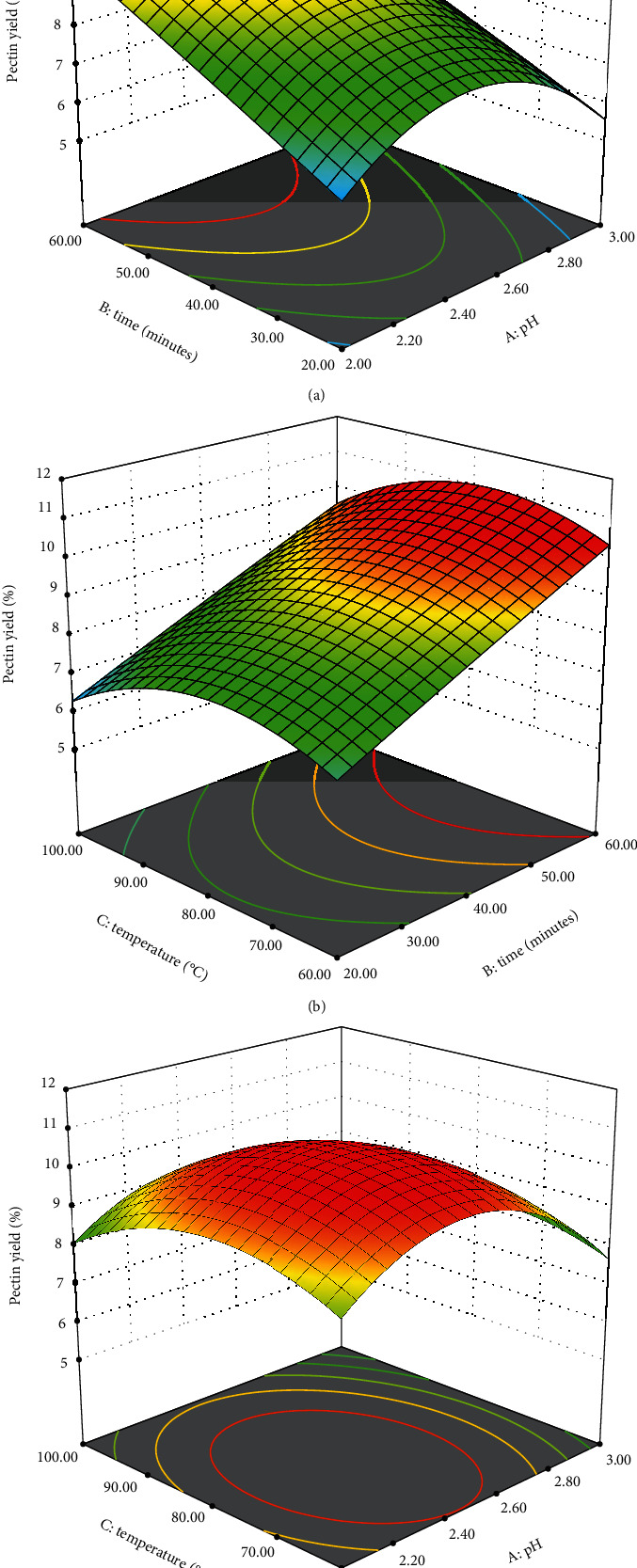
Effect of independent variables on pectin yield: (a) influence of pH and time, (b) influence of time and temperature, and (c) influence of pH and temperature.

The extraction conditions, including pH, time, and temperature, significantly influence pectin yield [31, 55]. In this study, the linear terms of all three variables demonstrated a statistically significant impact on the response variable (Table 3). pH stands out as an important factor in pectin extraction. Typically, a decrease in pH enhances pectin extraction yield, as the acidic conditions facilitate the hydrolysis of insoluble pectin into a soluble form, promoting higher yields [56]. However, beyond a certain limit, a further decrease in pH may lead to a decline in pectin yield, as highly acidic conditions can promote hydrolyzation, as observed in this study (Figure 3(a)). The pectin yield was maximum at pH 2.5, further increase and decrease impacted pectin yield. Similar findings have been reported by other studies as well [24, 57, 58].

The duration of extraction is a crucial factor in pectin extraction. In Figure 3(b), it is observed that the pectin yield from lotkon increased with time. While an increase in extraction time generally leads to higher yields, prolonged periods of extraction may result in decreased yields. Extended time in acidic conditions during extraction can lead to the breakdown of glycoside and ester bonds of pectin [59], impacting the overall yield and quality.

Temperature is also another key factor that influences pectin yield. Increased temperature contributes to higher cell disruption and enhanced solubilization. While the pectin yield may initially increase with a rise in temperature, reaching a certain level can lead to a subsequent decrease [60, 61]. Similarly, in this study, pectin yield was found to be highest at a temperature around 80°C; further increase in temperature decreases the yield (Figure 3(c)).

### 3.4. Optimization and Verification of the Optimum Condition

The optimal extraction conditions for pectin were determined using Derringer's desirability function approach in this study. The software “Design Expert” was employed, with the independent variables (pH, time, and temperature) ranging from 2 to 3 pH, 20 to 60 mins, and 60°C to 100°C, respectively. The sole response variable was the pectin yield, aiming to maximize its value. The software identified the optimal conditions as follows: pH (2.40), extraction time (56 min), and temperature (76°C), predicting a pectin yield of 10.73% with a maximum desirability of 1. To validate the optimal conditions, three extraction replicates were conducted with slight modifications (rounding) to the optimal values, resulting in pH (2.40), extraction time (56 min), and temperature (76°C). The experimental yield under these suggested optimum conditions was 10.56%, closely aligning with the predicted value. This agreement indicates the suitability of the derived model for predicting, optimizing, and maximizing the pectin yield from lotkon peel. The result found from this study is similar to other studies that used the conventional acid extraction method, such as 10.70% from cocoa pod husk [29], 11.62% from unripe ponkan mandarin [15], 14.60% from passion fruit peels [32], 14.60% from mango peel [34], slightly higher than 5.60% from Burdock [62], and 6.10%–9.00% from pomelo peels [30]. Hence, it is sufficient to say that the BBD has been an effective tool for the optimization of extraction conditions for maximizing pectin yield from lotkon peel.

### 3.5. Verification of Model Competency

**Figure 4:**
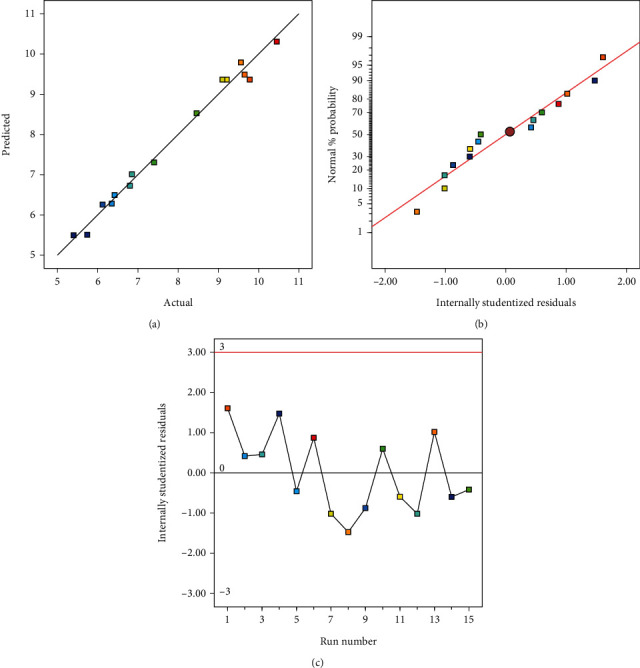
Diagnostic plots verifying model competency: (a) predicted versus actual values, (b) normal probability plot of residuals, and (c) internally studentized residuals across different runs.

The competency of the model was assessed through various diagnostic plots, including predicted versus actual, normal percentage probability, and internally studentized residuals, as illustrated in Figures 4(a), 4(b), and 4(c). The predicted values are closely aligned with the actual experimental values, forming a nearly straight diagonal line and indicating significant relevance to the actual data (Figure 4(a)). The normal percentage probability plot of residuals exhibited a routine dispersion, with residuals lying close to a straight diagonal line (Figure 4(b)). Additionally, the internally studentized residuals plot, presented in Figure 4(c), demonstrated an acceptable fit of the model, revealing that all data points fell within the ± 3*δ* margins.

### 3.6. Physiochemical Analysis of Dry Lotkon Peel Powder and Extracted Dry Pectin

The lotkon peel powder exhibited a moisture content of 9.75% (Table 4), comparable to values reported for banana dry peel, orange dry peel, citrus dry peel, and jackfruit dry peel (9.80%, 7.90%, 7.58%, and 6.48%, respectively) by Pathak, Mandavgane, and Kulkarni [63].

The moisture content of pectin derived from lotkon was 10.42% (Table 4), within the International Pectin Producers Association (IPPA) standard range of a maximum of 12.00% [61]. This value aligns with studies on pectin from citrange by Zouambia et al. [65] at 10.01%, from elephant apple and pomelo peels ranging from 6.98% to 9.04% by Rahman et al. [66], and higher than pectin from Moroccan *Citrus clementina* peels by Azzouzi et al. [55] at 12%, but lower than pectin from fruits of wild cardamom by Mbaku et al. [53] at 6.90%. Higher moisture content may compromise pectin quality by promoting microbial growth and pectinase enzyme production [67]. Additionally, moisture is a crucial parameter for meeting industry standards and plays a vital role in the shelf life of the product.

Water activity is a crucial measure for determining available free water that may facilitate microbial growth. The water activity of the peel powder and extracted pectin samples, measured at 0.49 and 0.51, respectively (Table 4), indicates levels low enough to inhibit the growth of microorganisms. However, it is worth noting that these values are slightly higher than the water activity of pectin from different berries, as reported by Muñoz-Almagro et al. [68], ranging from 0.32 to 0.40.

The ash content of lotkon peel powder was determined to be 5.36% (Table 4), well below the maximum limit of 10% set by IPPA [64]. This value is comparable to those reported for banana dry peel, orange dry peel, citrus dry peel, and jackfruit dry peel (5.01%, 5.25%, 4.32%, and 6.32%, respectively) by Pathak, Mandavgane, and Kulkarni [63], which are consistent with our findings. Maintaining a low ash content (below 10%) in pectin is indicative of higher purity and desirable functionality [69, 70]. In our investigation, the ash content of pectin extracted from lotkon was found to be 3.41% (Table 3), consistent with prior studies. Khamsucharit et al. [70] reported 3.46% ash content in citrus peel pectin, 1.96% in apple pomace pectin, and 1.38%–2.87% in banana peel pectin. Additionally, Rahman et al. [66] found a higher ash content of 7.07% in pectin from elephant apple, exceeding our findings in this study.

### 3.7. Characterization of Pectin

#### 3.7.1. Equivalent Weight

The equivalent weight of the extracted pectin from lotkon peel was measured as 769.23 mg/mole (Table 5), between the standard range by IPPA of 600–800 mg/mole [64]. The value is also comparable to the equivalent weight of apple pectin reported by Bhat et al. [71] at 725.24 mg/mole. Additionally, the equivalent weight ranged from 785.3 to 987.1 mg/mole for pectin extracted from elephant apple and pomelo fruit peels, as reported by Rahman et al. [66]. In comparison, our values are lower than apple pomace pectin (1666.30 mg/mole) but higher than cocoa husk pectin (645.19 mg/mole) as reported by Kumar and Chauhan [72] and Nazaruddin and Asmawati [73], respectively.

A higher equivalent weight contributes to stability and good emulsion capacity, with a recommended value of over 400 mg/mole for commercial pectin [66, 74]. The extracted pectin from this study demonstrated a significantly higher equivalent weight, indicating good quality.

#### 3.7.2. Methoxyl Content

The methoxyl content of the extracted pectin from lotkon peel was determined to be 7.75% (Table 5), slightly higher than commercial pectin reported by Pérez et al. [19] at 6.70%, but lower than pectin from watermelon rind at 10.66%. It falls well within the IPPA standard range of 2.2%–7.8% [64]. Additionally, it aligns with previously reported values for mango peel (7.33%), banana peel (7.03%), pomelo peel (8.57%), and passion fruit peel (8.81%–9.61%) [75]. Methoxyl content exceeding 7% classifies the pectin as high methoxyl (HM) pectin [76], indicating that the pectin derived in this study can be considered as HM pectin. Methoxyl content plays a crucial role in defining various functional properties of pectin, including gel strength, texture, setting time, sensitivity to metal ions, spreading quality, and sugar-binding capacity [26, 75]. Some studies have reported lower methoxyl contents, such as dragon fruit pectin with a methoxyl content of 2.98%–4.34% [69].

#### 3.7.3. Total Anhydrouronic Acid Content (AUA)

According to the Food Chemicals Codex [77], it is recommended that the galacturonic acid content (characterized as AUA) in commercial pectin should be above 65% as it indicates purity. Additionally, the IPPA specifies that the AUA of pectin should be a minimum of 35% [64]. The AUA of pectin derived from lotkon was found to be 66.88% (Table 5), thereby meeting the recommended purity threshold. This result aligns closely with the 68.10% of pectin from apple waste [71]. It is also higher compared to commercial apple pectin (61.72%) and dragon fruit pectin (45.25%–52.45%) as reported by Ismail et al. [69], as well as exceeding the values of 57.08% for pectin from pomelo peel and 54.03% for elephant apple pectin reported by Rahman et al. [66].

#### 3.7.4. Degree of Esterification (DE)

Commercially, pectin is characterized based on the DE. A DE value above 50% characterizes pectin as HM pectin, while a DE value below 50% indicates low methoxyl (LM) pectin, each with different applications [78, 79]. The extracted pectin from lotkon peel exhibited a DE of 65.79% (Table 5), similar to pectin from wild cardamom fruit at 66.61% [53], categorizing it as HM pectin. Other studies have reported higher DE values, such as 73.33% DE in watermelon rind pectin [19] and 83.41% DE in apple pomace pectin [80]. Conversely, some studies found lower values, such as Rahman et al. [66], who reported 56.95% for pectin from elephant apple.

#### 3.7.5. Acetyl Value

The acetyl values of lotkon peel were measured as 0.39% (Table 5), comparable to values reported for pectin from pumpkin peels (0.43%) by Hamed and Mustafa [81] and from a mixture of banana and papaya peels (0.48%) by Mada, Duraisamy, and Guesh [64]. Lower acetyl values are advantageous in the production of jams, jellies, and other food items, as acetyl groups can inhibit jelly formation [82]. Some studies have reported even higher acetyl values, such as Virk and Sogi [83], who found acetyl values of 0.62% for apple peel and 0.50% for commercial pectin.

### 3.8. Color Evaluation

In terms of color measurements, the lightness (*L*^∗^ values) of Lotkon peel pectin was recorded at 51.12, indicating a medium level of lightness. In comparison, Khamsucharit et al. [70] reported a higher lightness value for pectin extracted from banana peels. The redness (*a*^∗^ values) was lower, recorded at 17.05, while the yellowness (*b*^∗^ values) for lotkon peel pectin was recorded at 35.67, signifying less yellowishness in the pectin powder. The *L*^∗^ values and *b*^∗^ values are closely aligned with those observed by Masmoudi et al. [84] for lemon pectin derived from lemon by-products. However, the *a*^∗^ values were higher than those documented by Masmoudi et al. [84].

## 4. Conclusion

The present study successfully optimized the extraction of pectin from the peel of *Baccaurea ramiflora* Lour. (Burmese grape or lotkon) using an acidic extraction protocol. Utilizing the BBD of RSM, the optimal extraction conditions were determined to be a pH of 2.40, an extraction time of 56 min, and a temperature of 76°C. The actual pectin yield under these conditions was 10.56%, closely aligning with the model-predicted yield of 10.73%.

Characterization of the pectin extracted under these optimal conditions observed key quality parameters: moisture content of 10.42%, ash content of 3.41%, water activity of 0.51, equivalent weight of 769.23 mg/mole, methoxyl content of 7.75%, AUA of 66.88%, DE of 65.79%, and acetyl value of 0.39%. These findings demonstrate that the extracted pectin possesses excellent gelling, thickening, and stabilization capabilities, making it a viable alternative source for industrial pectin.

The study contributes not only to the efficient utilization of an underexploited fruit waste source but also expands the potential sources of pectin for industrial applications. Further investigation is recommended for scaling up the technology for bulk production of pectin employing this lab-scale knowledge. Additionally, exploring the applications and potential benefits of Burmese grape peel pectin in various industries, such as pharmaceuticals, cosmetics, and packaging, is recommended. This could pave the way for enhancing the value of Burmese grape peel as a sustainable and versatile resource.

## Figures and Tables

**Table 1:** Range and levels of the independent variables used in BBD.

**Range and levels of independent variables**			
**Variables**	**−1**	**0**	**+1**
A: pH	2.0	2.5	3.0
B: time (minute)	20	40	60
C: temperature (°C)	60	80	100

**Table 2:** Experimental runs of the extraction, with responses of actual and predicted pectin yield % (⁣^∗^center point).

**Run order**	**Independent variables**			**Pectin yield %**	
	**pH**	**Time (minute)**	**Temperature (°C)**	**Actual**	**Predicted**
1⁣^∗^	2.5	40	80	9.78	9.36
2	2.0	20	80	6.35	6.28
3	2.0	40	100	6.80	6.73
4	3.0	20	80	5.74	5.51
5	3.0	40	60	6.42	6.49
6	2.5	60	60	10.45	10.31
7⁣^∗^	2.5	40	80	9.10	9.36
8	2.0	60	80	9.56	9.79
9	2.5	20	100	6.12	6.26
10	2.0	40	60	7.40	7.31
11⁣^∗^	2.5	40	80	9.21	9.36
12	2.5	20	60	6.85	7.01
13	2.5	60	100	9.65	9.49
14	3.0	40	100	5.40	5.50
15	3.0	60	80	8.46	8.53

**Table 3:** ANOVA (analysis of variance) of the quadratic model derived for pectin yield from lotkon peel.

**Source**	**Sum of squares**	**d** **f**	**Mean square**	**F** ** value**	**p** ** value**	
Model	39.96	9	4.44	44.09	0.0003	Significant
A: pH	2.09	1	2.09	20.76	0.0061	
B: time	21.32	1	21.32	211.68	< 0.0001	
C: temperature	1.24	1	1.24	12.31	0.0171	
AB	0.06	1	0.06	0.596	0.475	
AC	0.0441	1	0.0441	0.4379	0.5374	
BC	0.0012	1	0.0012	0.0122	0.9165	
*A* ^2^	11.95	1	11.95	118.67	0.0001	
*B* ^2^	0.005	1	0.005	0.0493	0.8331	
*C* ^2^	4.14	1	4.14	41.13	0.0014	
Residual	0.5036	5	0.1007			
Lack of fit	0.2371	3	0.079	0.5933	0.6769	Not significant
*R* ^2^	0.9876					
Adjusted *R*^2^	0.9652					
Predicted *R*^2^	0.8914					
Adeq precision	18.5866					
CV %	4.06					

**Table 4:** Results of physiochemical analysis of dry lotkon peel powder and extracted dry pectin.

	**Lotkon peel powder**	**Lotkon peel pectin**
Moisture (%)	9.75 ± 0.87	10.42 ± 0.72
Water activity	0.49 ± 0.02	0.51 ± 0.03
Ash (%)	5.36 ± 0.74	3.41 ± 0.53

*Note:* All values given are means of three determinations (mean ± SD).

**Table 5:** Characterization of pectin extracted under optimal conditions.

**Characteristics**	**Lotkon peel pectin**
Equivalent weight (mg/mole)	769.23 ± 11.89
Methoxyl content (%)	7.75 ± 0.62
AUA (%)	66.88 ± 4.83
Degree of esterification (%)	65.79 ± 2.83
Acetyl value (%)	0.39 ± 0.04

*Note:* All values given are means of three determinations (mean ± SD).

## Data Availability

The data that support the findings of this study are available from the corresponding author upon reasonable request.
